# Developing Graphic Messages for Vaping Prevention Among Black and Latino Adolescents: Participatory Research Approach

**DOI:** 10.2196/29945

**Published:** 2021-11-23

**Authors:** Francisco Cartujano-Barrera, Chiamaka Azogini, Scott McIntosh, Maansi Bansal-Travers, Deborah J Ossip, Ana Paula Cupertino

**Affiliations:** 1 Department of Public Health Sciences University of Rochester Medical Center Rochester, NY United States; 2 Roswell Park Comprehensive Cancer Center Buffalo, NY United States

**Keywords:** vaping, electronic cigarettes, adolescents, Latino, Black

## Abstract

**Background:**

As an important transition stage in human development, adolescence is a critical window for vaping prevention. There is a substantial gap in communication research on vaping prevention among racial and ethnic minority groups. Their representation is essential to develop, implement, and disseminate innovative and effective interventions for vaping prevention.

**Objective:**

The aim of this study is to describe the participatory research (PR) procedures used with Black and Latino adolescents to develop culturally and linguistically appropriate graphic messages for vaping prevention.

**Methods:**

This PR study used a qualitative, user-centered design method. We conducted a series of focus groups with 16 Black and Latino adolescents to develop culturally and linguistically appropriate graphic messages for vaping prevention. The biobehavioral model of nicotine addiction provided a framework for the development of the graphic messages. Participants met 4 times to provide iterative feedback on the graphic messages until they reached a consensus on overall quality and content.

**Results:**

At baseline, the participants’ mean age was 15.4 years (SD 1.4). Of the participants, 50% (8/16) were female, 88% (14/16) were heterosexual, 56% (9/16) were Black/African American, and 44% (7/16) were Hispanic/Latino. A total of 12 of the 16 participants (75%) chose to participate in the English sessions. Participants decided to create four types of graphic messages: (1) financial reward, (2) health reward, (3) social norms, and (4) self-efficacy. Meeting 4 times with the 4 groups provided sufficient opportunities for iterative feedback on the graphic messages to reach a consensus on overall quality and content.

**Conclusions:**

It is feasible and practical to build PR among Black and Latino adolescents focused on vaping prevention. Adolescents added innovation and creativity to the development of culturally and linguistically appropriate graphic messages for vaping prevention. Appropriate staffing, funding, and approaches are key for successful PR efforts among Black and Latino adolescents. Future research is needed to evaluate the impact of the graphic messages on vaping prevention.

## Introduction

Adolescence (ages 10 to 19 years) [1] is a period characterized by tobacco use initiation, experimentation, and progression to long-term addicted use [2-5]. According to the US National Youth Tobacco Surveys, in 2018, 27.1% of high school students (an estimated 4.04 million) and 7.2% of middle school students (an estimated 840,000) reported current use of any tobacco product [6]. E-cigarettes were the most commonly used tobacco products among high school (20.8%) and middle school (4.9%) students [6]. In a cross-sectional survey conducted among a diverse sample of students in grades 7, 9, and 11 attending public schools in a low-income community in the United States, current vaping prevalence was 19% (176/963), compared to 6% (61/963) for cigarettes [7]. Moreover, in this same study, 55% of students were susceptible to future vaping, although there were no differences by race and ethnicity [7].

Although e-cigarettes *may* support smoking cessation [8-10], there is robust evidence that e-cigarette use during adolescence is associated with increased rates of future initiation of cigarette, alcohol, and marijuana use [11-13]. Moreover, early nicotine exposure puts adolescents at risk for a lifetime of vaping addiction as well as unknown health risks of long-term e-cigarette use. Chemical and heavy metal exposure from e-cigarettes and the risk of acute injuries and toxicity are a public health concern [14-19].

As an important transition stage in human development, adolescence is a critical window for tobacco prevention interventions to produce long-term reductions in the risk of cancer and other health outcomes [20]. Despite the adverse effects of vaping and the high prevalence and susceptibility of future vaping among adolescents, there is a lack of effective messages and communication channels to prevent initiation. To date, we know little about whether messages can prevent vaping among adolescents and if so, what messages and delivery formats may be most effective. One study found that chemical and anti-industry warning messages on television advertisements reduced intention to purchase e-cigarettes [21]. Another study found that addiction warnings increased the perceived harms and addictiveness of e-cigarettes and decreased thoughts about vaping when presented separately from advertising [22]. One limitation of both studies is the lack of representation of Black and Latino adolescents. This leaves a substantial gap in communication research for vaping prevention among racial and ethnic minority groups. Their representation is essential to develop, implement, and disseminate innovative and effective interventions for vaping prevention in the United States.

Participatory research (PR) is the coconstruction of research between researchers and people affected by the issues under study [23]. PR strengthens relations between the community and academia, ensures the relevancy of research questions, and increases the community’s capacity to identify and solve their problems [24,25]. PR seeks to move from conducting research “on” communities to conducting research “with” communities; as such, it represents a paradigm shift that better enables health research with underserved populations [26]. PR is an approach that has been used successfully among adolescents [27]. For tobacco prevention, environmentally oriented, youth-led programs have been identified as particularly effective in engaging youth [28,29]. However, to the best of our knowledge, no PR approach has been conducted for vaping prevention among Black and Latino adolescents. This manuscript describes our collaborative procedures with Black and Latino adolescents to develop graphic messages for vaping prevention.

## Methods

### Study Design

This PR study used a qualitative, user-centered design method. A user-centered design is typically defined as “a design that uses the natural properties of the individuals, exploiting the relationships and constraints and focusing on the needs and interests of the user, in order to make the final products usable and understandable” [30]. Consistent with some qualitative, user-centered design methodologies, we conducted a series of focus groups with 16 Black and Latino adolescents to develop culturally and linguistically appropriate graphic messages for vaping prevention. Participants met 4 times to provide iterative feedback on the graphic messages until they reached a consensus on overall quality and content. Participants received a US $25 gift card per session as a compensation for their time and effort. Study procedures were approved and monitored by the University of Rochester Medical Center Institutional Review Board (STUDY00005394).

### Conceptual Framework

A range of interacting multilevel factors influence tobacco use. This study is guided by the biobehavioral model of nicotine addiction, which recognizes the influence of social (eg, social norms, social support), psychological (eg, reward, self-efficacy), and biological factors (eg, depression, stress) in relation to tobacco use, cessation, and relapse [31]. These levels of influence also provide a framework for understanding how to most effectively reach and influence adolescents.

### Recruitment

Trained bilingual (English and Spanish), ethnically and racially diverse staff recruited Black and Latino adolescents. Consistent with community-based recruitment strategies [32], we relied on working with community-based organizations and public schools, and on word of mouth from participants. We provided two informational letters, via email, to individuals who were eligible to participate. One was addressed to the parents or caregivers and another to the adolescent. The letters, which were available in English and Spanish, described the study, explained the risks and benefits, and included the team contact information.

### Screening

Once the parents or caregivers received and reviewed the informational letter, they were instructed to contact the study team via phone call or email. Research staff then scheduled a phone call with interested parents or caregivers to determine the participant’s eligibility and ask them to verbally assent to their adolescent’s participation. During the phone call, research staff discussed all aspects of study participation and confidentiality, and they answered any questions. Parents or caregivers were informed that they and their adolescent were free to decline study participation and end their involvement at any time without negative consequences. The eligibility assessment and assent were available in English and Spanish.

### Eligibility

Individuals were eligible if they (1) self-identified as African American/Black and/or Hispanic/Latino, (2) were 12 to 17 years of age, (3) knew how to read and speak English and/or Spanish, (4) had never used e-cigarettes, (5) were willing to meet 4 times over the course of 8 weeks to develop and refine the graphic messages, and (6) had access to a device that was able to connect to web-based meetings.

### Assessments

Each participant completed the assessment in their language of preference. Assessments were adapted from surveys used in previous work and were pretested for survey administration among the research team [7]. The baseline survey collected information on demographics (eg, name, age, gender, sexual preference, and race), self-reported general academic performance, and smoking and vaping history of parents or caregivers.

### Procedures

Sessions were conducted via the institutional Zoom platform. Participants were sent a Zoom link via email with instructions to not forward or share the link with anyone else to ensure privacy. As an additional layer of security, each participant was held in a virtual “waiting room” until the moderating research staff allowed them into the meeting. Each session consisted of up to 4 participants who spoke the same language (either English or Spanish). A bilingual (English and Spanish), experienced researcher with extensive background in qualitative and PR methods (FCB) facilitated the sessions [33,34]. As a way to promote dialogue, participants were encouraged to turn on their cameras and ice breakers were incorporated at the start of each session. Icebreakers included questions like “If you could have a superpower, what would it be and why?”

For the first session, we conducted a brief presentation on general information regarding e-cigarette use. The presentation included operational definitions (eg, what is an e-cigarette and how does it work?), general statistics related to vaping use among middle and high school students, and health risks associated with vaping. In addition, we explained the biobehavioral model of nicotine addiction and defined it as the conceptual framework. After these preliminary actions to provide participants with a good working knowledge of the background information and the conceptual framework, we proceeded to elicit ideas for images and captions to include in the graphic messages for vaping prevention. A study team member (CA) took written notes during the first session. Participants’ suggestions were compiled into a general document and shared with a graphic designer who incorporated their ideas into coherent graphic messages.

For the second session, we presented prototype graphic messages to participants and prompted them to share their thoughts regarding the quality and content of the graphic messages. For sessions 3 and 4, we presented updated graphic messages to the participants. We prompted the participants again to share their thoughts regarding the quality and content of the updated graphic messages. A study team member (CA) took written notes for each of these sessions.

### Analysis

Frequencies were calculated for categorical variables. Means and standard deviations were calculated for continuous variables. The review team documented session notes, which were reviewed and organized through a process of abductive analysis. Abductive analysis is a process of iteratively going back and forth between theory and data to arrive at new insights that are both empirically and theoretically grounded [35]. The first and second authors independently analyzed written notes using content analysis, informed by elements of the biobehavioral model of nicotine addiction. The first and second authors met weekly and compared findings to identify similarities and differences. The last author joined for in-depth discussions if differences were found in analysis between the first and second authors.

## Results

### Baseline Characteristics

Table 1 describes the baseline characteristics of the participants. At baseline, the participants’ mean age was 15.4 years (SD 1.4), 50% (8/16) were female, 88% (14/16) were heterosexual, 56% (9/16) were Black/African American, and 44% (7/16) were Hispanic/Latino. A total of 12 of the 16 participants (75%) chose to partake in the English sessions, while 4 participants (25%) chose the Spanish sessions. Over half of the participants (9/16, 56%) were in the 11th grade. Half of the participants (8/16, 50%) reported having very good overall academic performance. A total of 2 participants (13%) reported that at least one parent or caregiver used tobacco products.

**Table 1:** Baseline characteristics of participants (N=16).

Characteristics		Value
Age (years), mean (SD)		15.4 (1.4)
**Gender, n (%)**		
	Female	8 (50)
	Male	8 (50)
**Sexuality, n (%)**		
	Heterosexual	14 (88)
	Bisexual	1 (6)
	Prefer not to answer	1 (6)
**Race, n (%)**		
	Black or African American	9 (56)
	American Indian or Alaska Native	2 (13)
	Multiracial	2 (13)
	Other	3 (19)
**Ethnicity, n (%)**		
	Hispanic or Latino	7 (44)
	Not Hispanic or Latino	9 (56)
**Language used in the focus group session, n (%)**		
	English	12 (75)
	Spanish	4 (25)
**Current grade level, n (%)**		
	8	4 (25)
	9	1 (6)
	10	2 (13)
	11	9 (56)
**Overall academic performance, n (%)**		
	Very good	8 (50)
	Good	7 (44)
	Average	1 (6)
**Use of tobacco products among parent or caregiver, n (%)**		
	Neither parent smokes tobacco-related products	14 (88)
	At least one parent or caregiver uses tobacco-related products	2 (13)

### Development of the Graphic Messages

Meeting 4 times with the 4 groups provided sufficient opportunities for iterative feedback on the graphic messages to reach a consensus on overall quality and content. Participants decided to create four types of graphic messages: (1) financial reward, (2) health reward, (3) social norms, and (4) self-efficacy. Participants engaged in robust conversations. The discussions sometimes led to a finite conclusion regarding participants’ recommendations for the graphic messages (eg, changing the background color or rewording a particular phrase in the caption). However, in the instances where there were multiple ideas still being debated, the team opted to have the graphic designer create multiple options for the participants to vote on in the subsequent meeting. Below is a description of each of the graphic messages and the creation process.

#### Financial Reward

**Figure 1:**
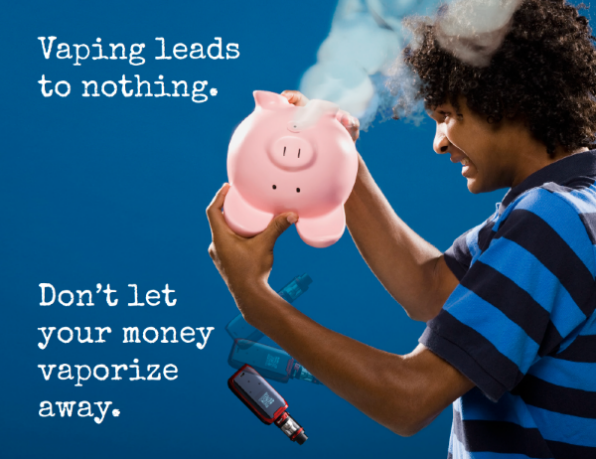
Graphic message on the topic of financial reward.

Participants decided to create a message about the economic benefits of not vaping. They proposed a graphic message showing a teenage boy looking at a piggy bank, wondering where his money went. Participants asked that the piggy bank have e-cigarette aerosol coming out of its mouth and an e-cigarette falling out of it. They decided that the best caption was “Vaping leads to nothing. Don’t let your money vaporize away” (Figure 1). For the graphic message in Spanish, participants decided not to use the term “*vapear*” (Spanish for “vaping”) but to use the term “*usar cigarrillos electrónicos*” (Spanish for “use electronic cigarettes”). Spanish-speaking participants mentioned they had never heard the term “*vapear*” and that it did not sound correct to them.

#### Health Reward

**Figure 2:**
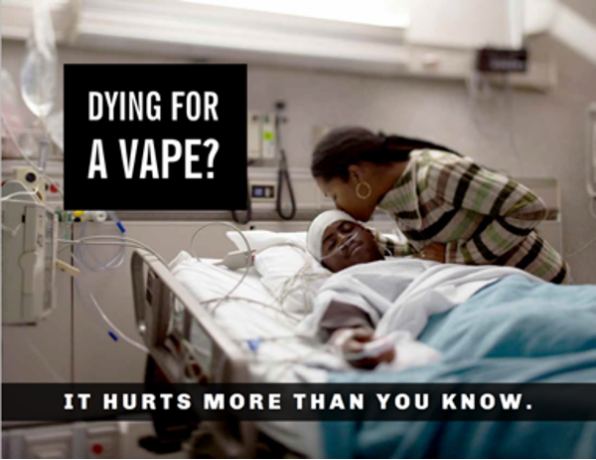
Graphic message on the topic of health reward.

Participants decided to create a message about the health consequences of vaping. Rather than developing a graphic message to scare teenagers by showing the negative health effects of vaping, they decided to develop a message that resonates with their emotions. Participants were surprised to learn that, as of February 2020, vaping has been connected to 2807 lung injury cases and 68 deaths in the United States [36]. They decided that one way to raise awareness of these alarming numbers was to elucidate the family impact of lung injuries and deaths caused by vaping. They suggested the graphic message show a mother kissing her son who is hospitalized in the intensive care unit. Participants expressed that the idea of not letting their mothers down was a very strong emotional deterrent for vaping. They decided the best caption was “Dying for a vape? It hurts more than you know” (Figure 2).

#### Social Norms

**Figure 3:**
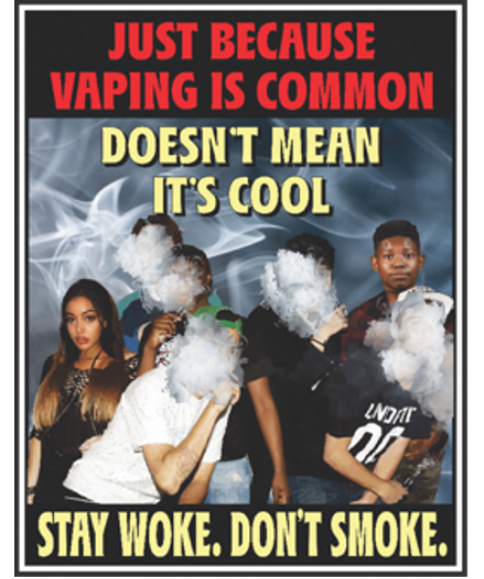
Graphic message on the topic of social norms.

The study participants acknowledged the social pressures related to vaping initiation and decided to create a message about changing the social acceptability of vaping. They wanted the graphic message to show a group of Black and Latino teens standing together, with some holding e-cigarettes in their hands and e-cigarette aerosol covering their faces. They also wanted to include two teens without e-cigarettes, but with smoke around their faces to exemplify the underlying message of standing against the social influences of vaping among peers. For the caption, the participants agreed that “Just because vaping is common, doesn’t mean it’s cool” was an easily understandable and appropriate message to include. However, they wanted the message to feel more lighthearted and relatable to their peers. Therefore, they identified a captivating and culturally relevant phrase of “Stay woke” and suggested adding the additional phrase of “Don’t smoke” to heighten the appeal of the overall message (Figure 3). Spanish-speaking participants recognized that the phrase “Stay woke. Don’t smoke” does not have a literal translation. So, they decided to use the phrase “*Ponte alerta. No fumes*” (Spanish for “Stay alert. Don’t smoke”).

#### Self-efficacy

**Figure 4:**
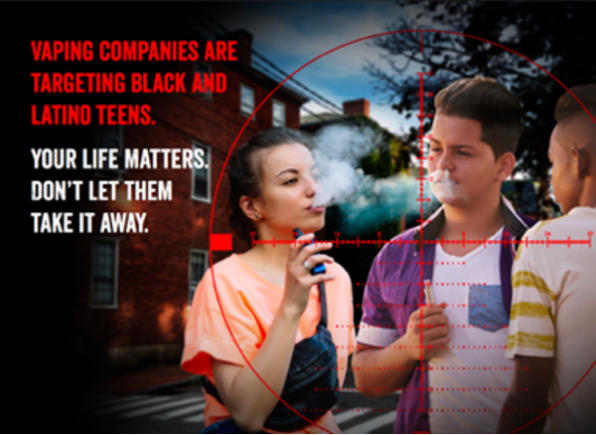
Graphic message on the topic of self-efficacy.

Participants were particularly surprised to learn about the racial and ethnic disparities in vape shop density. Vape shops are more densely distributed in neighborhoods with large Black and Latino populations [37,38]. They expressed that a message empowering other adolescents with the knowledge of these disparities would be useful to avoid “falling into their trap.” The participants proposed a graphic message showing Black and Latino adolescents being targeted by a sniper’s scope. They suggested the graphic message include both a girl and a boy to be more inclusive. Participants decided the best caption was “Vaping companies are targeting Black and Latino teens. Your life matters. Don’t let them take it away.” They noted that people often use the terms “Black” and “African American” interchangeably. They decided to use the term “Black” in the graphic messages as it better represented their reality and contains less characters, which would make the message less wordy. Although the Spanish translation of the term “Black” is “*Negro,*” Spanish-speaking participants decided to use the term “*Afroamericano*” (African American) in the Spanish messages to avoid being offensive. Participants also noted that people often use the terms “Hispanic” and “Latino” interchangeably. However, they decided to use the term “Latino” in the graphic messages as, from their perspective, it is broader and refers to people, music, and culture. Both English- and Spanish-speaking participants were unfamiliarized with the gender-neutral terms “Latinx” and “Latine.” So, they decided to use the term “Latino,” as it “sounds nicer” and is a term that unites all Latinos in general. Participants suggested using the sentence “Your life matters” in light of the Black Lives Matter movement (Figure 4).

### Retention

All participants (16/16, 100%) attended the first session. A total of 15 of the 16 participants (94%) attended the second session, and 13 participants (81%) attended the third and fourth sessions.

## Discussion

### Principal Results

This study demonstrated that it is practical to build PR among Black and Latino adolescents focused on vaping prevention. Moreover, this work demonstrated that it is feasible to recruit and retain Black and Latino adolescents, two traditionally hard-to-reach groups, into a tobacco control study. Partnering with community-based organizations and public schools was a key strategy for recruiting participants. Furthermore, race, ethnicity, and language matching of research staff to subjects was likely important for approaching both parents and adolescents for study participation.

This study provided Black and Latino adolescents, who are frequently underrepresented in tobacco prevention research, with an opportunity to participate in the development of graphic messages for vaping prevention. In addition to gaining exposure to research, participants were provided with an empowering space to develop a sense of purpose and possibility. Far from looking at Black and Latino adolescents as “at-risk” or in need of knowledge, we welcomed them into our research as experts, as voices from the community needed to improve our practice, and as colleagues. Within this environment, adolescents may become conscious of their realities and envision themselves as agents of change capable of transforming their communities. Moreover, as described in the US Centers for Disease Control and Prevention’s *Best Practices User Guide for Youth Engagement in Tobacco Prevention and Control*, young people naturally challenge the traditional attitudes that may limit how adults think and act [39]. In this study, adolescents added innovation and creativity to the development of graphic messages for vaping prevention. Critical to this PR effort was the provision of monetary compensation for participants. As described by Ozer [40], monetary compensation increases youth’s feelings of equity, value, and investment in research projects.

This study adds valuable insights to the expanding literature on PR among adolescents from underrepresented minority groups. This PR approach with Black and Latino adolescents has the potential to be replicated in research involving other public health concerns. Engaging youths through PR can be successful for developing, implementing, and interpreting the results of research and can offer the potential to overcome the challenges of developing culturally and linguistically appropriate, community responsive interventions for underserved populations [41]. Moreover, as elucidated by Patchen et al [42], PR integrated with user-centered design methods offers practical, culturally relevant, and systematic guidance to develop appropriate interventions for underserved groups. Lastly, the use of abductive analysis provided a comprehensive method for developing graphic messages that were both empirically and theoretically grounded.

### Future Work

The next steps of this project include conducting a pilot randomized controlled trial (RCT) with 360 Black and Latino adolescents to evaluate the impact of the graphic messages for vaping prevention. The participants will be randomized to receive 1 of the 4 graphic messages (ie, self-efficacy, social norms, health reward, and financial reward). Through the pilot RCT, we will assess immediate postexposure measures of susceptibility to future vaping. Study measures of susceptibility to future vaping will be comprised of valid items [21,22]. We will assess three items: (1) Are you curious about using e-cigarettes (vaping)? (2) Do you think that you will use e-cigarettes (vaping) in the next 12 months? and (3) If one of your best friends offered you an e-cigarette, would you use it? Responses include (1) definitely not, (2) probably not, (3) probably yes, and (4) definitely yes. The goal of this future pilot RCT is to provide preliminary data for future studies, including the possible effect size.

### Conclusions

It is feasible and practical to build PR among Black and Latino adolescents focused on vaping prevention. Adolescents added innovation and creativity to the development of culturally and linguistically appropriate graphic messages for vaping prevention. Appropriate staffing, funding, and approaches are key efforts to PR among Black and Latino adolescents. Future research is needed to evaluate the impact of the graphic messages for vaping prevention.

## Abbreviations

FDA: Food and Drug Administration
NIH: National Institutes of Health
PR: participatory research
RCT: randomized controlled trial
